# Participation in school sports among children and adolescents with juvenile idiopathic arthritis in the German National Paediatric Rheumatologic Database, 2000–2015: results from a prospective observational cohort study

**DOI:** 10.1186/s12969-019-0306-9

**Published:** 2019-02-11

**Authors:** Florian Milatz, Jens Klotsche, Martina Niewerth, Nils Geisemeyer, Ralf Trauzeddel, Elisabeth Weißbarth-Riedel, Tilmann Kallinich, Joachim Peitz, Matthias Hartmann, Kirsten Minden

**Affiliations:** 1 Epidemiology Unit, German Rheumatism Research Centre Berlin, Chariteplatz 1, 10117 Berlin, Germany; 2 Department of Paediatric Rheumatology, Helios Klinikum Berlin-Buch, Schwanebecker Chaussee 50, 13125 Berlin, Germany; 3 Paediatric Rheumatology Clinics, University Hospital Eppendorf, Martinistraße 52, 20251 Hamburg, Germany; 4 Department of Pediatrics, Division of Pneumonology and Immunology with intensive Medicine, Charité University Medicine Berlin, Augustenburger Platz 1, 13353 Berlin, Germany; 5 Paediatric Rheumatology Centre, Asklepios Clinic, Sankt Augustin, Arnold-Janssen-Straße 29, 53757 Sankt Augustin, Germany; 6 German Centre for Paediatric and Adolescent Rheumatology, Gehfeldstraße 24, 82467 Garmisch-Partenkirchen, Germany; 7 Epidemiology Unit, German Rheumatism Research Centre Berlin and Department of Rheumatology and Clinical Immunology, Charité University Medicine Berlin, Chariteplatz 1, 10117 Berlin, Germany

**Keywords:** Juvenile idiopathic arthritis, School sports, Prevalence, Correlates, Trends, Children and adolescents, Participation

## Abstract

**Background:**

Regular school sports can help adolescents achieve the recommended amount of daily physical activity and provide knowledge, attitudes and behavioral skills that are needed in order to adopt and maintain a physically active lifestyle. Furthermore, it reaches all children including those that are at risk for engaging in more sedentary types of behavior. Since adolescents with juvenile idiopathic arthritis (JIA) are less involved in physical and social activities than their healthy peers, the objectives were to (1) estimate the prevalence of participation in school sports among patients with JIA; (2) determine the correlates associated with school sports absenteeism; and (3) investigate whether attendance in school sports has changed in the era of biologics.

**Methods:**

Data from schoolchildren with JIA recorded in the German National Paediatric Rheumatologic Database (NPRD) in the years 2000 to 2015 were considered for the analyses. Data from the year 2015 were inspected to analyze correlates of school sports absenteeism. Whether school sports participation had changed between 2000 and 2015 was determined using linear mixed models.

**Results:**

During the 15-year period, the participation rates in school sports were determined in 23,016 patients. The proportion of patients who participated in school sports almost always steadily increased from 31% in 2000 to 64% in 2015 (β = 0.017, 95% confidence interval (CI) 0.015, 0.020), whereas the exemption rate simultaneously decreased from 44% in 2000 to 16% in 2015 [β = − 0.009, 95% CI -0.011, − 0.007]. In 2015, the data from 5879 patients (mean age 13.1 ± 3.3 years, female 65%, disease duration 5.9 ± 4.0 years, persistent oligoarthritis 37%) were available for evaluation. Full exemption from school sports (in 16.1% of cases) was associated with functional limitations, disease activity and any use of DMARDs, intra-articular glucocorticoid injections or physiotherapy.

**Conclusions:**

School sports attendance among children and adolescents with JIA has increased significantly over the past 15 years. Possible explanations include improved functional ability probably due to better treatment options. The integration of patients with child acceptable symptom states who have previously been fully exempted from school sports needs to be addressed in the future.

## Background

Juvenile idiopathic arthritis (JIA) is the most common chronic inflammatory rheumatic disease in childhood with an annual incidence in Western countries of 2–20 patients per 100,000 and a prevalence of 16–150 patients per 100,000 [1]. Defined by seven categories, JIA comprises a clinically heterogeneous group of autoimmune inflammatory diseases of unknown origin that begin before the age of 16 and last for at least 6 weeks [2, 3]. The most common accompanying symptoms mediated by chronic inflammation are significant impairments, such as persistent pain, limited joint motion and fatigue, which may contribute to a reduced health-related quality of life and impaired social functioning [4–6].

Apart from drug therapy, lifestyle modifications play an important therapeutic adjunct to reduce symptoms frequently associated with JIA. In this regard, recent research suggests the importance of physical activity, which is associated with significant improvements in physical and mental health in both healthy children and those with JIA [7–10]. Participation in these activities can be accompanied by improved body composition, bone mineral density and emotional factors, thereby positively affecting disease-specific symptoms, such as pain [11], swollen joints [12] and reduced quality of life [8, 9, 13].

To maintain or achieve activity-induced health benefits over the long term, current WHO physical activity guidelines recommend that children and adolescents should engage in at least 60 min of moderate to vigorous physical activity (MVPA) daily [14]. In this context, a substantial proportion of both healthy children and especially those with JIA seem to be not active enough to benefit from their age-appropriate fitness level [15, 16].

The school environment facilitates opportunities for physical activity that reaches all children including those that are at risk for engaging in more sedentary types of behavior [17]. Recent findings suggest that school sports are important sources for MVPA engagement in boys and girls [18]. Further, it has been mentioned that children do not compensate for a sedentary school day by increasing their levels of physical activity after school [19]. Moreover, school sports can provide knowledge, attitudes and behavioral skills that adolescents need in order to adopt and maintain a physically active lifestyle [20, 21]. Since childhood is described as the ideal time to develop motor skills and establish social competence, school sports can also provide health benefits by positively affecting musculoskeletal development and psychological outcomes, such as self-concept, self-esteem and social behavior [21].

Due to a lack of data concerning school sports in children with JIA, this is the first study providing information on the prevalence, trends and correlates of participation in school sports among children and adolescents with JIA. Cross-sectional data from the National Paediatric Rheumatologic Database (NPRD) in Germany were used.

## Methods

### Patients

The NPRD of children and adolescents with rheumatic diseases started nationwide in 1997 and covers a wide-ranging spectrum of juvenile rheumatic diseases. The data on the disease phenotypes and outcome measures are prospectively collected using standardized physician and patient questionnaires. These questionnaires are filled out during the routine consultation once per year. The number of participating rheumatology centres increased from 27 in 2000 to more than 60 in 2015 and now includes all major centres in Germany. The most frequent diagnosis documented each year is JIA (approximately 75% of cases). According to estimates of the JIA prevalence, the about 9000 recorded cases correspond to almost 70% of the expected JIA cases in Germany. For more details regarding this representative database including sociodemographic and clinical characteristics, as well as treatment assignments, of children and adolescents with rheumatic diseases, see Minden et al. [22–24]. The study was approved by the Charité - Berlin Medical University Ethics Committee.

The inclusion criteria for the analyses of school sports attendance in the presented study were as follows: 1) diagnosis of JIA according to the International League of Associations for Rheumatology (ILAR) criteria [2], 2) enrollment in the database between 2000 and 2015 and 3) school attendance. The year 2000 was chosen because patient school sports attendance has been recorded since that year.

### Measures

The sociodemographic and clinical characteristics reported by the paediatric rheumatologist include the patient’s age, sex, diagnosis, age at disease onset, disease duration, and laboratory values, such as erythrocyte sedimentation rate (ESR), body height and weight. BMI was calculated as the weight in kilograms divided by the height in meters squared. Additionally, the physician evaluated the patient’s disease activity (physician’s global assessment, PGA) on a numerical rating scale (NRS; from 0 = no disease activity to 10 = very severe disease activity). Inactive disease was defined as a physician’s global assessment of disease activity score of 0.

The patient-reported outcomes on an NRS included an evaluation of pain, fatigue, coping and overall well-being.

The patients aged ≥13 years or the parents of patients aged < 13 years reported on their functional ability using the German version of the Childhood Health Assessment Questionnaire (C-HAQ) [25]. The resulting disability index ranges from 0 to 3, whereby a value of zero indicates no functional disability and higher scores indicate light, moderate or severe level of disability. The clinical Juvenile Arthritis Disease Activity Score in 10 joints (cJADAS-10) [26] was developed as a composite tool for scoring disease activity. The clinical JADAS-10 considers the number of joints with active disease and the physician’s and patient’s/parent’s global assessment without considering the ESR. Child acceptable symptom states were defined as minimal disease activity. In accordance with Consolaro et al. [27], the JADAS cutoff for classification of minimal disease activity was 2 for oligoarticular JIA and 3.8 for polyarticular JIA.

The frequency of physical activity in leisure time is a patient-reported variable on a five-point Likert scale, ranging from ‘daily’ to ‘3-5 times a week’, ‘1-2 times a week’, ‘more seldom’, and ‘never’. The patients aged ≥13 years or the parents of patients aged < 13 years also reported parental vocational training ranging from ‘academic degree’ to ‘apprenticeship’ and ‘no training’.

### Treatment

The rheumatologist recorded treatment with glucocorticoids (GCs), conventional synthetic disease-modifying anti-rheumatic drugs (csDMARDs), and biologic DMARDs (bDMARD) within the past 12 months. Medication with systemic GCs comprises the categories of low dose (< 0.2 mg/kg body weight/day), high dose (≥ 0.2 mg/kg/day), and intravenous pulse therapy. Additional intra-articular GC injections were reported. With respect to patient-reported treatment, the following 4 categories of non-pharmacological treatment were provided: physiotherapy, kinetotherapeutic bath, occupational therapy and no non-pharmacological treatment.

### School sports attendance

In Germany, participation in school sports is generally compulsory throughout the school career. All students are expected to participate unless with a medical exemption. Participation in school sports was reported by the patients aged ≥13 years or the parents of patients aged < 13 years) on a four-point Likert scale ranging from ‘almost always’ to ‘sometimes not’, ‘often not’, and ‘exempt from school sports’.

### Statistical analyses

The categorical variables were reported by absolute and relative frequencies, whereas continuously distributed variables were reported by means and standard deviations (95%-confidence interval (CI)). The annual school sports attendance was determined for the years 2000 to 2015 in the NPRD for the total cohort and for JIA categories. The change in school sports attendance between 2000 and 2015 was analyzed by two-level random effect logit models. The sociodemographic and clinical parameters were included in the logit models to investigate the association of school sports attendance with these parameters. A *p*-value less than 0.05 was considered to be statistically significant. All the statistical analyses were performed using SAS 9.3 (SAS Institute Inc., Cary, NC, USA).

## Results

### Sociodemographic information and clinical characteristics

Between 2000 and 2015, the number of school-aged patients with JIA included in the database ranged from 1463 (in 2000) to 5879 (in 2015). During this period, the patient characteristics did not differ relevantly in terms of female sex, age, BMI, and disease duration. The clinical data as well as the self-reported outcomes recorded in the years 2000, 2003, 2006, 2009, 2012, and 2015 are presented in Table 1.

**Table 1:** Characteristics of school-aged JIA patients recorded in the years 2000, 2003, 2006, 2009, 2012, and 2015

	2000	2003	2006	2009	2012	2015
No. of patients	1463	2339	3020	3526	3722	5879
Age, years	12.3 ± 3.2	12.6 ± 3.3	12.7 ± 3.4	12.6 ± 3.3	13.7 ± 2.5	13.1 ± 3.3
BMI, kg/m2	─	19.4 ± 3.8	19.4 ± 3.9	19.4 ± 4.8	19.7 ± 2.8	19.1 ± 3.2
Female, no. (%)	898 (61.4)	1464 (62.4)	1901 (62.9)	2246 (63.7)	2374 (63.8)	3844 (65.4)
Disease duration, years	4.9 ± 3.6	5.5 ± 3.3	5.3 ± 3.5	5.1 ± 3.3	5.5 ± 3.6	5.9 ± 4.0
Age at disease onset, years	7.4 ± 4.1	7.7 ± 4.2	7.7 ± 4.3	7.8 ± 4.2	8.5 ± 4.2	7.9 ± 4.4
JIA category, no. (%)						
RF-positive polyarthritis	51 (3.5)	59 (2.5)	65 (2.2)	99 (2.8)	114 (3.1)	145 (2.5)
RF-negative polyarthritis	206 (14.1)	9 (12.3)	461 (15.3)	495 (14.0)	645 (17.3)	1084 (18.4)
Systemic JIA	105 (7.2)	127 (5.4)	150 (5.0)	164 (4.7)	128 (3.4)	264 (4.5)
Persistent oligoarthritis	526 (36.0)	943 (40.2)	1238 (41.0)	1637 (46.4)	1479 (39.7)	2165 (36.9)
Extended oligoarthritis	140 (9.6)	157 (6.7)	180 (6.0)	225 (6.4)	349 (9.4)	652 (11.1)
Psoriatic arthritis	133 (9.1)	254 (10.8)	275 (9.1)	234 (6.6)	225 (6.0)	342 (5.8)
Enthesitis-related arthritis	196 (13.4)	400 (17.0)	528 (17.5)	570 (16.2)	664 (17.8)	1052 (17.9)
Unclassified JIA	106 (7.2)	110 (4.7)	123 (4.0)	102 (2.9)	118 (3.2)	175 (2.9)
cJADAS-10	5.8 ± 5.5	4.3 ± 4.7	4.3 ± 4.8	4.0 ± 4.6	4.0 ± 4.7	4.0 ± 4.7
PGA score^φ^	1.7 ± 1.7	1.3 ± 1.7	1.4 ± 1.7	1.3 ± 1.7	1.4 ± 1.8	1.2 ± 1.8
Inactive disease*, no. (%)	429 (30.0)	952 (41.4)	1229 (41.3)	1444 (42.6)	1423 (39.8)	3130 (54.6)
No. of joints with active disease	3.2 ± 5.7	1.6 ± 3.4	1.6 ± 3.6	1.5 ± 3.3	1.3 ± 3.2	1.1 ± 2.9
ESR, mm/h	10.7 ± 15.1	11.7 ± 12.5	12.6 ± 14.6	11.7 ± 12.6	10.1 ± 10.7	11.2 ± 12.8
C-HAQ total score	0.3 ± 0.5	0.2 ± 0.4	0.2 ± 0.4	0.2 ± 0.4	0.2 ± 0.4	0.2 ± 0.4
No functional limitations†, no. (%)	738 (51.3)	1300 (55.8)	1717 (58.8)	2151 (61.4)	2280 (61.8)	3465 (59.9)
Patient-reported well-being^φ^	1.8 ± 2.1	1.7 ± 2.0	1.7 ± 2.0	1.6 ± 2.0	1.6 ± 2.1	1.8 ± 2.2
Patient-reported pain^φ^	1.6 ± 2.3	1.6 ± 2.2	1.6 ± 2.2	1.6 ± 2.3	1.7 ± 2.5	1.8 ± 2.5
Patient-reported fatigue^φ^	─	─	─	─	1.5 ± 2.5	1.5 ± 2.5
Patient-reported coping^φ^	1.6 ± 0.9	1.6 ± 0.9	1.4 ± 2.1	1.4 ± 2.0	1.3 ± 2.1	1.4 ± 2.1
Treatment in past 12 months, no. (%)						
No systemic GCs	1103 (78.8)	1768 (81.3)	2323 (85.2)	2535 (88.5)	2683 (89.4)	4519 (92.4)
Low-dose GCs (< 0.2 mg/kg)	244 (17.4)	336 (15.4)	343 (12.6)	246 (8.6)	258 (8.6)	236 (4.8)
Combination of high-dose GCs/						
pulse therapy	10 (0.7)	11 (0.5)	14 (0.5)	16 (0.5)	19 (0.5)	29 (0.5)
Intra-articular GCs	255 (18.2)	195 (9.0)	299 (11.0)	317 (11.1)	261 (8.7)	434 (8.9)
Any conventional synthetic DMARD	674 (48.9)	1013 (48.6)	1496 (56.0)	1672 (54.7)	1861 (58.0)	1828 (30.7)
Any biologic DMARD	11 (0.8)	114 (11.3)	295 (9.8)	432 (14.1)	650 (20.6)	1140 (22.3)
Regular exercise in leisure time (patients aged ≥13)‡, no. (%)	─	─	2174 (72.9)	2677 (77.1)	3029 (82.9)	2409 (79.1)
Physical therapies, no. (%)						
Physiotherapy	972 (67.9)	1299 (56.8)	1645 (55.9)	1714 (50.4)	1844 (51.0)	2830 (49.9)
Kinetotherapeutic bath	132 (9.2)	110 (4.8)	130 (4.4)	123 (3.6)	96 (2.7)	147 (2.6)
Occupational therapy	119 (8.3)	200 (8.7)	316 (10.7)	318 (9.3)	381 (10.5)	567 (10.0)
No physical therapy	401 (28.0)	903 (39.5)	1177 (40.0)	1557 (45.7)	1679 (46.5)	2687 (47.4)

*JIA* juvenile idiopathic arthritis *RF* rheumatoid factor, *cJADAS-10* 10-joint clinical Juvenile Arthritis Disease Activity Score, *PGA* physician’s global assessment, *ESR* erythrocyte sedimentation rate, *C-HAQ* Childhood Health Assessment Questionnaire; GC, glucocorticoid, *DMARD* disease-modifying antirheumatic drug

*Defined by a PGA score of zero

†Defined by a C-HAQ score of zero

^φ^Assessed on a numerical rating scale (maximum score 10) by patients aged ≥13

‡‘daily/3–5 times a week/1–2 times a week’ vs. ‘less than once a week/never’

Approximately two-thirds of the sample were female. The mean ± SD age of patients at the onset of JIA was a minimum of 7.4 ± 4.1 years in 2000 and a maximum of 8.5 ± 4.2 years in 2012. The proportion of patients with persistent oligoarthritis documented in this study differed over the course of the 15 years, characterized by 36.0% in 2000 to 46.4% in 2009 and 36.9% in 2015. The use of biologic DMARDs increased between 2000 and 2015 (from 1% in 2000 to approximately 22% in 2015). With respect to the use of systemic glucocorticoids, a decrease was observed starting in 2000 (21.2%) and reaching 7.6% in 2015. The proportion of patients who participated in leisure time-based physical exercise at least once a week increased relevantly from 2006 to 2015.

### School sports attendance in JIA patients from 2000 to 2015 and changes over time

**Fig. 1:**
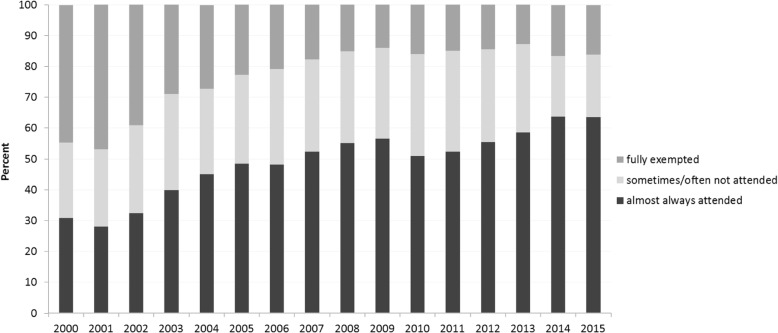
School sports attendance and exemption rates in children and adolescents with JIA during the period 2000–2015. *significant time effect (attendance, *p* < 0.001; exemption, *p* < 0.001), adjusted for JIA category, disease duration, age at disease onset and sex

**Fig. 2:**
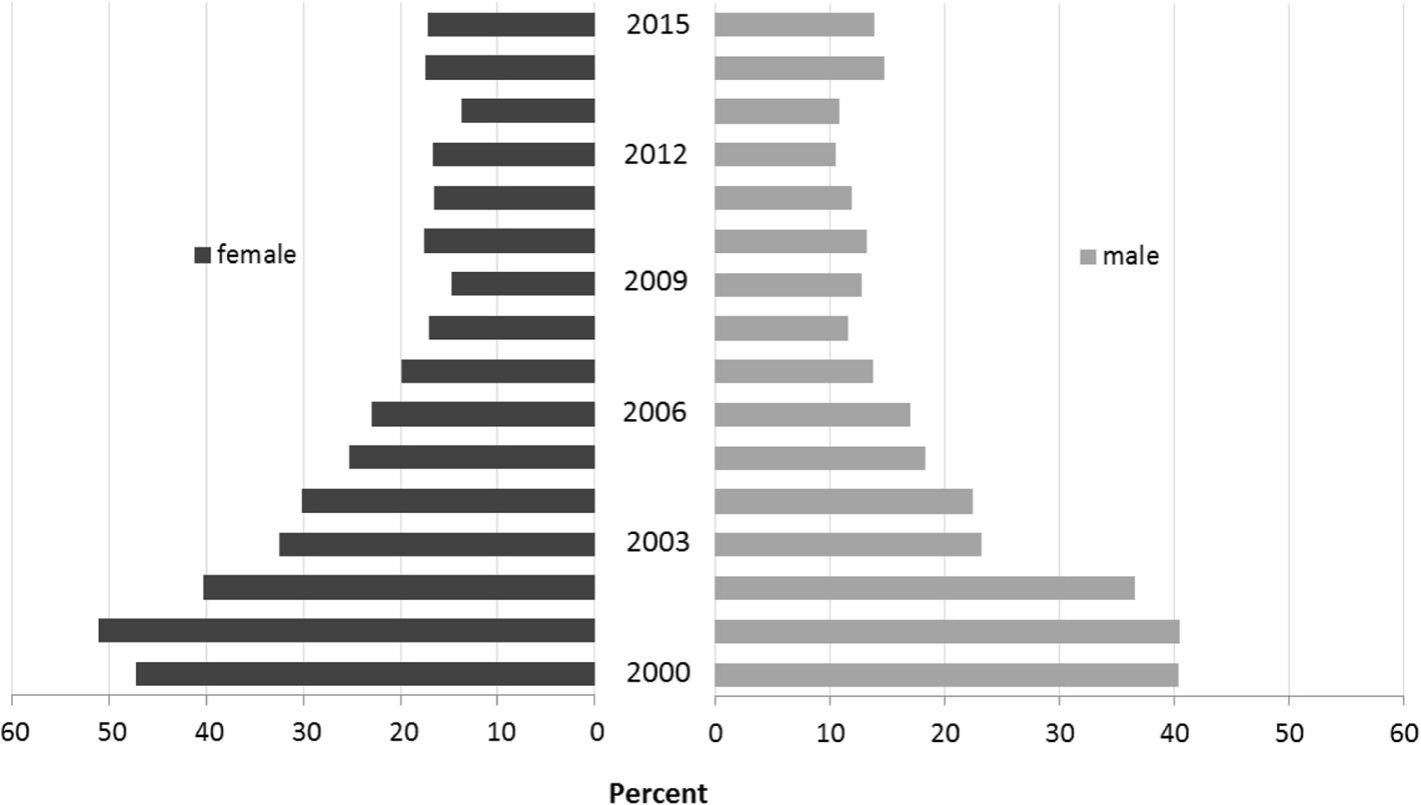
Sex-specific rates of full exemption from school sports in JIA patients during the period 2000–2015. *significant time effect (female, *p* < 0.001; male, *p* < 0.001)

The participation rates recorded between 2000 and 2015 in school sports were determined in 23,016 children and adolescents with JIA (51,676 cases). The proportion of patients who participated in school sports almost always increased significantly from 30.8% in 2000 to 63.6% in 2015 (β = 0.017, 95% CI 0.015, 0.020), mainly caused by a significant decrease in the rate of patients who were fully exempted from school sports (from 44.5% in 2000 to 16.1% in 2015; β = − 0.009, 95% CI -0.011, − 0.007). Additionally, the proportion of patients who reported participating sometimes or often in school sports simultaneously decreased slightly over the same period from 24.6% in 2000 to 20.2% in 2015 (Fig. 1). The decrease in the exemption rate was associated with an increasing proportion of both patients with inactive disease (β = 0.02, 95% CI 0.01, 0.02) and patients without functional limitations (β = − 0.13, 95% CI -0.14, − 0.10) over time. Females (OR 1.3, *p* < 0.007) were more likely to be fully exempted from school sports than were boys (Fig. 2). Moreover, full exemption from school sports was associated with both GCs (β = 0.13, 95% CI 0.09, 0.17) and physical therapy (β = 0.14, 95% CI 0.13, 0.16) over time.

Full participation in school sports was significantly associated with increased treatment of biologic (β = 0.002, 95% CI 0.00, 0.01) DMARDs, reduced functional limitations (β = 0.217, 95% CI 0.195, 0.239) and disease activity (β = − 0.012, 95% CI -0.014, − 0.009) as well as less severe self-reported pain (β = − 0.027, 95% CI -0.033, − 0.022).

The largest decreases in the exemption rate were observed in patients with RF-positive polyarthritis (from 67.3% in 2000 to 25.2% in 2015; *p* < 0.001) and systemic JIA (from 52.4% in 2000 to 16.7% in 2015; *p* < 0.001). The use of systemic GCs in patients with RF-positive polyarthritis and systemic JIA decreased significantly from 61.7% in 2000 to 20.9% in 2015 and 75.0% in 2000 to 31.8% in 2015, respectively. The decreasing use of systemic GCs as well as lowered functional limitations between 2000 and 2015 was associated with a higher attendance rate in school sports in both RF-positive polyarthritis and systemic JIA patients. This association was also true after adjusting for age, sex and disease duration. Additionally, the decreasing use of GCs in these categories was associated with an increasing use of biologic agents.

### Correlates of exemption from school sports in 2015

In 2015, 63.6 and 20.2% of patients participated almost always and sometimes in school sports, respectively, whereas 16.1% were fully exempted. The variables associated with exemption from school sports are shown in Table 2.

**Table 2:** Univariate and multivariable correlates of exemption from school sports in the 5879 patients recorded in the year 2015

Variable	Univariate			Multivariable		
	OR	95% CI	*P*	OR	95% CI	*P*
Age	1.11	1.09–1.14	< 0.001	1.09	1.04–1.14	< 0.001
BMI	1.07	1.05–1.10	< 0.001	1.01	0.97–1.05	0.656
Female sex	1.30	1.11–1.52	0.001	1.00	0.78–1.28	1.000
Disease duration	0.99	0.99–1.00	0.049			
Age at disease onset	1.08	1.06–1.10	< 0.001	1.03	1.00–1.06	0.041
RF-positive polyarthritis	1.60	1.13–2.26	0.008	1.25	0.74–2.13	0.407
RF-negative polyarthritis	0.98	0.83–1.16	0.831	0.73	0.55–0.97	0.028
Systemic JIA	0.96	0.71–1.29	0.777	1.44	0.88–2.37	0.147
Persistent oligoarthritis	0.80	0.69–0.92	0.003	1.36	1.07–1.73	0.013
Extended oligoarthritis	1.07	0.88–1.31	0.487	1.29	0.95–1.76	0.097
Psoriatic arthritis	1.16	0.90–1.50	0.248	1.11	0.74–1.65	0.618
Enthesitis-related arthritis	0.85	0.71–1.02	0.075	0.71	0.52–0.96	0.028
cJADAS-10	1.15	1.13–1.17	< 0.001	1.08	1.03–1.13	0.001
PGA score	1.33	1.29–1.38	< 0.001			
Inactive disease*	0.28	0.24–0.33	< 0.001			
No. of joints with active arthritis	1.10	1.08–1.12	< 0.001			
ESR	1.02	1.02–1.03	< 0.001			
C-HAQ total score	1.13	1.11–1.15	< 0.001	1.85	1.39–2.45	< 0.001
No functional limitations†	0.24	0.21–0.28	< 0.001			
Overall well-being^φ^	1.30	1.27–1.34	< 0.001			
Pain intensity^φ^	1.25	1.22–1.28	< 0.001	1.04	0.97–1.11	0.265
Fatigue^φ^	1.17	.14–1.20	< 0.001	0.97	0.92–1.02	0.235
Coping^φ^	1.28	1.24–1.32	0.001	1.05	0.98–1.13	0.191
Treatment in past 12 months						
Any systemic GCs				1.35	0.91–2.01	0.137
Low-dose GCs (< 0.2 mg/kg)	2.06	1.43–2.96	< 0.001			
Any high-dose GCs (> 0.2 mg/kg)≠	1.90	1.36–2.67	< 0.001			
Intra-articular GCs	1.97	1.55–2.51	< 0.001	1.52	1.08–2.15	0.017
Any DMARD	1.67	1.42–1.97	< 0.001	1.70	1.33–2.19	< 0.001
Parent with academic degree	1.31	1.02–1.67	0.035			
Regular exercise in leisure time (patients aged ≥13)‡	0.46	0.38–0.57	< 0.001			
Physiotherapy	1.88	1.62–2.18	< 0.001	1.27	1.01–1.60	0.040
Occupational therapy	1.54	1.24–1.92	< 0.001	0.96	0.66–1.39	0.819
No physical therapy	0.55	0.47–0.64	< 0.001			

*OR* odds ratio, 95% CI 95% confidence interval, *RF* rheumatoid factor, *JIA* juvenile idiopathic arthritis, *JADAS-10* 10-joint Juvenile Arthritis Disease Activity Score, *PGA* physician’s global assessment, *ESR* erythrocyte sedimentation rate, *C-HAQ* Childhood Health Assessment Questionnaire, *GCs* glucocorticoids, *DMARD* disease-modifying antirheumatic drug

* Defined by a PGA score of zero

† Defined by a C-HAQ score of zero

^φ^ Assessed on a numerical rating scale (maximum score 10)

≠ Defined as high dose GCs (≥ 0.2 mg/kg) and/or intravenous pulse therapy

‡ ‘daily/3–5 times a week/1–2 times a week’ vs. ‘less than once a week/never’

**Fig. 3:**
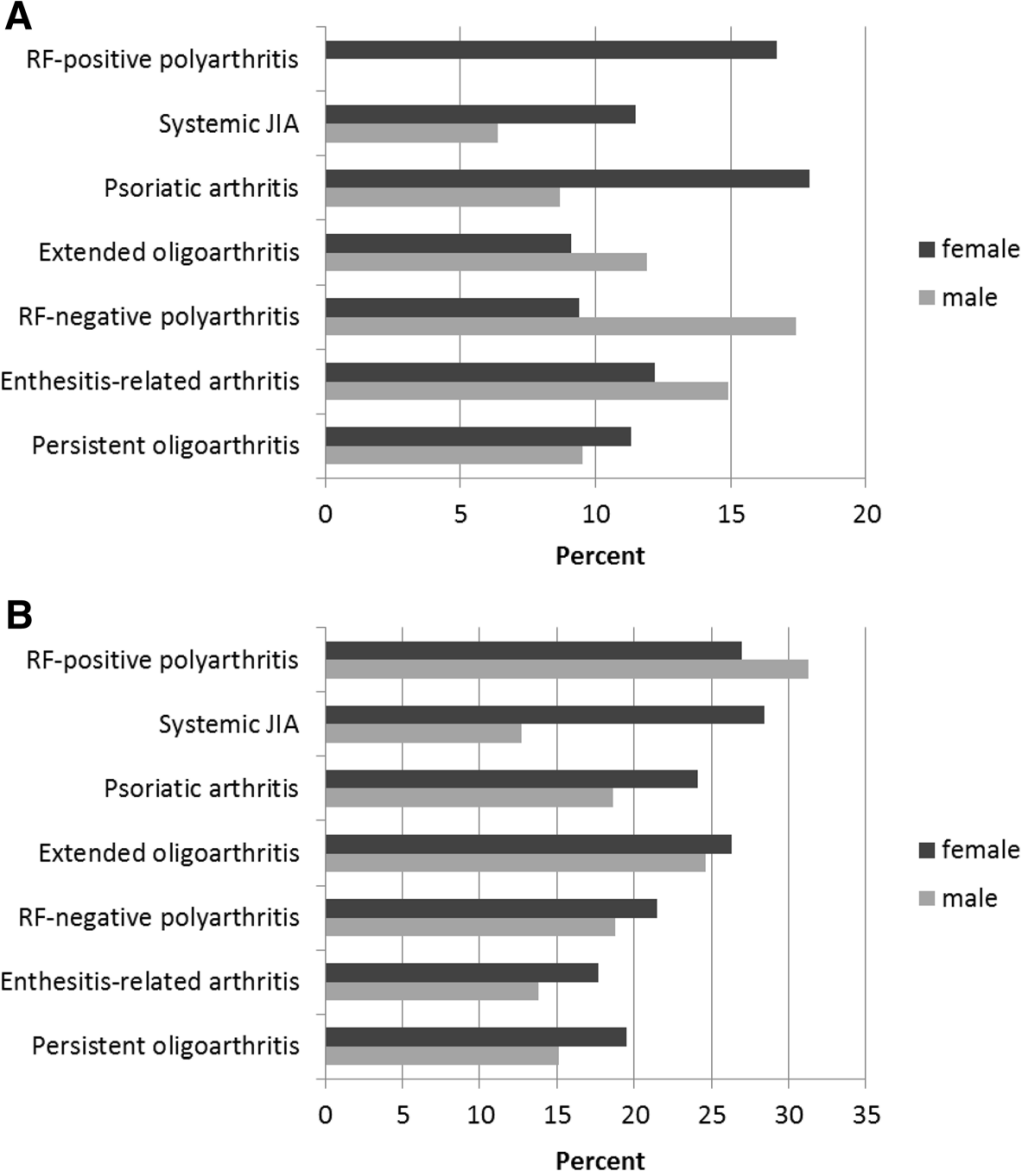
Sex-specific rates of full exemption from school sports by JIA category in the year 2015. **a**) patients aged under 13, **b**) patients aged 13 and above

In the multivariate analyses, the patients with persistent oligoarthritis were more likely to be exempted, whereas patients with RF-negative polyarthritis and enthesitis-related arthritis had higher attendance rates. As shown in Fig. 3, the sex-specific exemption rates significantly differed within the JIA categories. The exemption rates were higher in females, in patients reporting higher functional limitations according to the C-HAQ (C-HAQ score > 0), and in patients with a higher disease activity as well as in patients who received any GCs, DMARDs or who received physical therapy. Higher age, higher age at disease onset, and lower physical activity in leisure time were associated with exemption from school sports in the univariate analyses. Moreover, self-reported pain, fatigue, coping and overall well-being were associated with increased risk of non-participation.

## Discussion

The results of this study extend the current literature by providing the prevalence, trends and correlates of young patients with rheumatic disease and attendance in school sports, which were recently suggested as important sources for MVPA engagement in boys and girls [18]. Increasing age, functional limitations, higher disease activity, intra-articular GCs, DMARDs and physiotherapy were associated with exemption from school sports. Also, JIA patient participation in school sports steadily increased during the period of 2000–2015, whereas the proportion of patients who were fully exempted simultaneously decreased. Finally, the sharpest decrease in the exemption rate was found in patients with RF-positive polyarthritis and systemic JIA, which was associated with increased functional capacity and lower disease activity.

To our knowledge, this is the first study investigating the participation in school sports among children and adolescents with JIA over a long time period using a large database. We have shown that increased biologic DMARD use, improved functional capacity and lowered disease activity are associated with a decreasing exemption rate. Achieving inactive disease status and reducing functional limitations are among the major goals of treatment of JIA. The proportion of patients with inactive disease (physician’s global assessment of zero) and without functional limitations increased significantly between 2000 and 2015, which reflects overall improvements in the treatment of patients with JIA over the last 15 years. In 2015, more than 50% of patients achieved a state of inactive disease at documentation, probably resulting in higher attendance rates in school sports and leisure-time physical activity, as shown in our study. However, since school sports is an important source for MVPA in children [18], and programs to increase MVPA have the potential to reduce disease-specific symptoms [11–13, 28–30], it might also be possible that the observed increase in school sports attendance supports the achievement of a clinically inactive state in a subgroup of patients.

In Germany, students normally receive up to 135 min of compulsory school sports per week. This movement time is particularly important for those who tend to have a pronounced sedentary lifestyle and higher risks of developing comorbidities.

Although school sports alone cannot achieve the recommended amount of daily physical activity, one hour of school sports increased the activity levels of girls on school days by approximately 31% and those of boys by 45% [31]. In this context, Dale and colleagues [19] have shown that children cannot compensate for an inactive school day by engaging in out of school activities in the afternoon. Since there is some evidence in young children with rheumatic disease suggesting that physical inactivity may have deleterious effects in the disease manifestation by deteriorating physical capacity and muscle function [32], regular participation in school sports may contribute, at least in part, to reducing disease burden.

Interestingly, recent findings from Schenck et al. [23] noted that physical inactivity is not simply a result of functional limitations, and that inactive patients may avoid physical activity despite their ability to participate. Taking into account the existing cut-off values [27] and referring to our data, 8% of patients with persistent oligoarthritis and 11% of patients with rheumatoid factor-negative polyarthritis are fully exempt from school sports despite being in a child acceptable symptom state. Although physical exercise has increasingly become part of the JIA treatment protocol, other studies have also shown no correlation between the level of physical activity and the disease severity [33, 34]. However, it cannot be ruled out that concomitant pain syndromes or other reasons rather than an active JIA were responsible for the non-participation in some patients.

With regard to variables associated with school sports attendance, our results are in line with recent findings by Armbrust et al. [35], which revealed both being off medication and lower disability as significant predictors of full participation in school sports.

In our study, less severe self-reported pain was associated with full attendance in school sports. This result is comparable to that of previous groups [36, 37], which found physical activity to be inversely related to pain. Prior studies evaluating participation in school settings additionally demonstrated that adolescents with pain conditions may consequently feel less accepted and less supported by both peers and school staff [38]. Furthermore, there is evidence suggesting that these adolescents report more difficulty with peer relationships than healthy peers and are more isolated and withdrawn, are less well liked and have fewer reciprocal friendships [39]. Since regular participation in school sports can promote the development of social skills, social behaviors and self-esteem [40], school sports may contribute to mental health and psychosocial well-being in school life.

Recent work by Schanberg et al. [41] revealed that children and adolescents with JIA are at a greater risk for adopting a more sedentary lifestyle compared to their healthy peers in part due to disease-related factors such as fatigue. In our study, self-reported fatigue was not associated with exemption from school sports (after adjustment), which indicates that factors other than fatigue contribute more to absenteeism from school sports.

Referring to our findings regarding the demographic data, boys showed higher participation rates in school sports than girls, and younger children showed higher attendance rates than older children. These findings are in line with studies from Maggio et al. [42] and Takken et al. [43], where these variables were significantly associated with higher leisure time physical activity. However, since leisure-based exercise and school sports are not comparable, our results probably cannot be directly compared to those of previous groups.

Patients who were fully exempted from school sports were more likely to be treated with GCs. However, the overall use of GCs decreased over time. Rescuing patients from the need for long-term treatment with GCs is significant because of the associated risks of serious side effects, including excessive weight gain, osteoporosis, fractures, arterial hypertension and premature atherosclerosis, as seen in the studies by Raab et al. [44] and Aulie et al. [45].

Our findings must be interpreted with caution in light of several potential limitations. The participation in school sports was a question directed to the patient and parents and was not verified by schools. Additionally, no national data are available that show population-level trends over time in weekly school sports attendance. Therefore, we cannot consider our findings in relation to healthy peers.

## Conclusions

In conclusion, an increasing interest in lifestyle modifications as long-term treatment options in patients with JIA with previously reported low physical activity levels suggests the need to search for effective ways to increase MVPA and, therefore, potentially reduce disease-specific symptoms and risks for developing comorbidities. The participation in school sports steadily increased between 2000 and 2015. Better functional capacity and lower disease activity, mainly due to earlier and more frequent use of DMARDs, were associated with school sports attendance. Since an increased MVPA is associated with a decreased total sedentary time, and school sports is an important source for MVPA engagement, regular participation in school sports may play a role in disability reduction and the improvement of social functioning in JIA. Future work should address the integration of patients with child acceptable symptom states who have previously been exempt from school sports. In this context, the implementation of individualized medical certificates, which allow disease-adjusted participation, might be useful. Therefore, it will be necessary to continue to provide comprehensive information to teachers, parents and physicians regarding the opportunities and risks of school sports. Whether the material recently distributed by the German Society for Paediatric and Adolescent Rheumatology (GKJR) will lead to increasing sports participation will be seen in the near future.

## Abbreviations

bDMARDs: Biologic disease-modifying anti-rheumatic drugs
BMI: Body mass index
C-HAQ: Childhood health assessment questionnaire
CI: Confidence interval
cJADAS-10: 10-joint clinical Juvenile Arthritis Disease Activity Score
csDMARDs: Conventional synthetic disease-modifying anti-rheumatic drugs
ESR: Erythrocyte sedimentation rate
GC: Glucocorticoid
ILAR: International League of Associations for Rheumatology
JADAS: Juvenile arthritis disease activity score
JIA: Juvenile idiopathic arthritis
MVPA: Moderate-to-vigorous physical activity
NPRD: German National Paediatric Rheumatologic Database
NRS: Numeric rating scale
OR: Odds ratio
WHO: World Health Organization
